# Effect of High Hydrostatic Pressure Extraction on Biological Activities and Phenolics Composition of Winter Savory Leaf Extracts

**DOI:** 10.3390/antiox9090841

**Published:** 2020-09-08

**Authors:** Sílvia A. Moreira, Sara Silva, Eduardo Costa, Soraia Pinto, Bruno Sarmento, Jorge A. Saraiva, Manuela Pintado

**Affiliations:** 1 LAQV-REQUIMTE, Departamento de Química, Universidade de Aveiro, 3810-193 Aveiro, Portugal; silvia.moreira@ua.pt (S.A.M.); jorgesaraiva@ua.pt (J.A.S.); 2 CBQF—Centro de Biotecnologia e Química Fina—Laboratório Associado, Escola Superior de Biotecnologia, Universidade Católica Portuguesa, 4169-005 Porto, Portugal; sara.nc.silva@gmail.com (S.S.); emcosta@porto.ucp.pt (E.C.); 3 Nanomedicines and Translational Drug Delivery, Instituto de Investigação e Inovação em Saúde, Universidade do Porto 208, 4200-135 Porto, Portugal; soraia.pinto@i3s.up.pt (S.P.); bruno.sarmento@ineb.up.pt (B.S.)

**Keywords:** winter savory, high-pressure extraction, phenolic compounds, antioxidant activity, cytotoxicity

## Abstract

*Satureja montana* L. has several biological properties related to its diverse composition of secondary metabolites. Nevertheless, it has been mainly studied for its essential oil, with only a few studies on the profile and bioactivities of the bioactive compounds from its leaf extracts being reported. This work aimed to study the antioxidant activity (by oxygen radical absorbance capacity (ORAC) assay), antimicrobial minimum inhibitory and bactericidal concentrations (MIC and MBC) determination, antibiofilm (by colorimetry), impact upon DNA (anti- and pro-oxidant assay), and cytotoxicity (by cell metabolism viability assays) of *S. montana* extracts obtained by high-pressure-assisted extraction (HPE). The extract obtained at 348 MPa, 35% (*v*/*v*) ethanol presented the highest concentration of individual phenolic compounds, and a minimum bactericidal concentration of 20 mg/mL against *Listeria monocytogenes*. HPE extracts showed antioxidant activity not only in ORAC but they were also able to prevent/attenuate peroxide-induced damage upon DNA. Moreover, on its own, HPE extract induced less oxidative damage than the control extract. Concerning the cytotoxicity, HPE extracts (at 0.5 and 1.0 mg/mL) were not harmful to HT29 cell lines, while control extracts (obtained at atmospheric pressure) at higher concentrations (>1.0 mg/mL) slightly reduced the metabolism of the cells. Finally, all extracts showed inhibition of the viability of 3 cancerous cell lines (>2.0 mg/mL for Caco-2, HeLa, and TR146) to below 15%.

## 1. Introduction

There is a growing consumer demand for “natural” food additives to be used as preservatives, antioxidants, colors, and flavors, as alternatives to synthetic ones. This is leading to an increment of studies related to the biological activities of plant extracts (from leaves, flowers, seeds, or fruit), especially on antioxidant and antimicrobial properties [1]. The biological activities, mainly the antioxidant activity of plant extracts, is usually attributed to the presence of phenolic compounds (phenolic acids, catechins, and flavonoids), whose antioxidant properties are generally due to redox properties (such as free-radical scavenging activity or transition-metal chelating activity) [2].

*Satureja montana* L., commonly known as winter savory or mountain savory, belongs to the Lamiaceae family, and it is a perennial herb usually found in the Mediterranean area such as southern Europe and North Africa and likes to grow in arid, sunny, and rocky regions [2,3]. Although it has been used as a culinary herb (as a spice for food and tea, mainly for seasoning of meat and fish, as well as a flavoring for soup, sauces, or canned food) since antiquity, it is used in traditional and homeopathic medicine to treat several ailments, such as digestive problems, such as colics and diarrhea, urinary tract complaints, or respiratory diseases such as bronchitis, cough, and also as an expectorant and anti-catarrhal [3,4,5,6]. The whole plant can be used for its medicinal benefits since this herb is rich in several biologically active constituents such as essential oils, tannins, triterpenes, and flavonoids [3,6,7,8]. Research studies in the literature report that its extracts have several biological activities, such as anti-inflammatory, antimicrobial, antifungal, antioxidative, and antiproliferative [9,10,11].

Typically, the essential oils are the most studied extracts obtained from winter savory, existing already an International Standard (ISO 7928-1-1991), which states that the minimum yield of essential oil (on a dry basis) required for springs of *S. montana* is 0.3% and it must contain *γ*-terpinene, *p*-cymene, linalool, 1-terpinen-4-ol, and carvacrol as main constituents [5]. Nevertheless, this kind of extract is usually obtained by hydrodistillation, requiring the use of high temperatures, and leading, consequently, to several problems of thermal degradation, oxidation, and hydrolysis of some compounds. High hydrostatic pressure assisted extraction (HPE) appears as an alternative method, where the mentioned limitations can be overcome, since it can operate at room temperature, avoiding the degradation of thermo-sensitive compounds [12]. HPE is also characterized as being environmentally friendly and having great potential in the food and pharmaceutical industries [13]. In recent years, HPE has been used for the extraction of carotenoids, ginsenosides, phenolic acids, flavonoids, anthocyanins, among others, from a diversity of food products, such as fruit, vegetables, food by-products, and herbs [14,15]. Concerning the use of HPE as a technology to extract bioactive compounds in savory, an optimization study was performed previously [16], and it was possible to obtain three sets of optimal conditions, depending on what one would want to extract: (i) for optimization of extraction yield and antioxidant activity (by 2,2-diphenyl-1-picrylhydrazyl (DPPH) assay), it was obtained a set of optimal conditions of 500 MPa, 20 min, 0% ethanol; (ii) total phenolic compounds, total flavonoids, antioxidant activity (by 2,2′-azino-bis(3-ethylbenzothiazoline-6-sulfonic acid) diammonium salt (ABTS) assay), and antioxidant activity (by ferric reducing antioxidant power (FRAP) assay) were optimized for 348 MPa, 20 min, 35% ethanol; and (iii) extraction of pigments (chlorophylls and carotenoids) was optimized for extraction at 500 MPa, 1 min, 70% ethanol [16].

Therefore, the present work aimed to study the effect of HPE on the biological activities of dried leaves winter savory extracts, previously obtained by optimization by response surface methodology [16]. HPE effect was evaluated on the individual compounds profile, antioxidant activity, antimicrobial, and antitumoral activity, as well as on the DNA oxidation capability and cytotoxicity of the final extracts. This study aimed to optimize the extraction process, to obtain final quality extracts, with a high concentration of bioactive compounds and improved biological activities, and, consequently, enhance the knowledge of an aromatic herb and promote its commercialization and valorization as a medicinal plant.

## 2. Material and Methods

### 2.1. Chemical Materials

Sodium dihydrogen phosphate and hydrogen peroxide were acquired from Merck (Darmstadt, Germany) and fluorescein, potassium phosphate dibasic, AAPH (2,20-azo-bis-(2-methylpropionamidine)-dihydrochloride), trolox (6-hydroxy-2,5,7,8-tetramethylbroman-2-carboxylic acid), DNA (deoxyribonucleic acid salt) from calf thymus, bromophenol blue sodium salt, and phenazine methosulfate solution (PMS) were purchased from Sigma Chemical (St. Louis, MO, USA). Agarose and GreenSafe Premium were purchased from Nztech (Lisboa, Portugal). Iron(III) chloride (FeCl_3_) was acquired from Panreac (Barcelona, Spain) and Tris-Acetate EDTA (ethylenediaminetetraacetic) (TAE) buffer was purchased from Grisp (Porto, Portugal). Caucasian colon carcinoma (Caco-2) and mucous producing human colon (HT29-MTX-E12) cells were obtained from the European Collection of Authenticated Cells Cultures (ECACC 8601020 and 12040401, respectively) through Sigma-Aldrich (St. Louis, MO, USA; ECACC) (references 09042001 and 12040401, correspondingly). DMEM (Dulbecco’s Modified Eagle’s Medium), Pen-Strep, and non-essential amino acids 100× were purchased from Lonza (Basel, Switzerland) and Fetal Bovine Serum (FBS) was obtained from Biowest (Nuaillé, France).

### 2.2. Biological Samples and Extraction Conditions

Winter savory leaves were offered by Cantinho das Aromáticas (Vila Nova de Gaia, Portugal), which were cultivated as an organic labeled product. To obtain the extracts, high hydrostatic pressure (HPE) was applied according to a previous work in which the optimization of the extraction conditions is described [16]. For so, HPE was carried out at room temperature (20–25 °C), using different extraction times (1–20 min), different solvents (0% (*v*/*v*) ethanol (aqueous), 35% ethanol:water, *v*:*v*, and 70% ethanol:water, *v*:*v*), and different pressure levels (348 and 500 MPa). Extracts were obtained by placing 1.0 g of fresh material in a container together with 10 mL of extraction solvent. Samples were homogenized with an Ultraturrax T25 homogenizer (Janke & Kunkel IKA-Labortechnik, Staufen, Germany), and the mixture was then placed in low permeability polyamide–polyethylene bags (Albipack-Packaging Solutions, Águeda, Portugal) that were heat-sealed manually with care to leave the minimum amount of air inside the bags. HPE experiments were carried out on an industrial scale high-pressure equipment (Hiperbaric 55, Hiperbaric, Burgos, Spain). Control samples were maintained at atmospheric pressure (0.1 MPa) under the same conditions of pressure, time, and solvent concentration. Controls were kept in the dark and surrounded by water to mimic all the conditions of the samples under pressure, except for the high pressure. After extraction, each sample was centrifuged at 5030× *g*, 10 min, at 4 °C (Heraeus Biofuge Stratos, Thermo, Electron Corporation, MA, EUA). The supernatant was then filtered using a 10–13 μm filter (Whatman n° 1 equivalent, 1250 Filter-Lab, Filtros Anoia, S.A., Barcelona, Spain), and the filtrates were collected and stored at −80 °C until further analyses. All extracts were performed in triplicate, and its nomenclature is described in Table 1.

### 2.3. Total Phenolic Compounds

The total phenolic compounds of the extracts were quantified using the Folin-Ciocalteu method [17]. Folin-Ciocalteu reagent (100 µL) was mixed with 20 µL of extract in a microplate with 96 wells. After resting for 4 min, 75 µL of sodium carbonate solution were added and the mixture rested for 2 h in the dark, at room temperature; the absorbance was registered at 750 nm (Multiskan Go microplate spectrophotometer, Thermo Fisher Scientific Inc., Waltham, MA, USA). Gallic acid was used as standard (0–200 mg/L) and the results were expressed as gram of gallic acid equivalents per 100 g of leaves dry weight (g_GAE_/100 g).

### 2.4. LC-MS/MS Analysis of Phenolic Compounds

Phenolics identification and quantification were conducted on an ultra-high performance liquid chromatograph (UHPLC) Ultimate 3000, Dionex liquid chromatography coupled to an Ultra-High Resolution quadrupole-time-of-flight (UHR-QqTOF) mass spectrometry (Impact II™, Bruker, Billerica, MA, USA, EUA). Mobile Phase: Eluent A: ultrapure water (100%, *v*/*v*) (Millipore system) with 0.1% (*v*/*v*) formic acid (Sigma-Aldrich, Germany), and eluent B: acetonitrile (100% (*v*/*v*)) (Merck pure grade) with 0.1% (*v*/*v*) formic acid; at a flow rate of 0.25 mL/min. The following gradient was employed: 0–10 min (0% eluent B); 10–14 min (21% eluent B); 14–18.3 min (27% eluent B); 18.3–20 min (58% eluent B); 20–21.5 min (95% eluent B) and 21.5–22 min (0% eluent B). Each run took 21 min to complete. The capillary voltage of the electrospray ionization (ESI) was set to 2500 V and the capillary temperature was 200 °C. Spectra were recorded in negative-ion mode between *m/z* 20 and 1000. Identification of individual phenolics (see Table 2) was carried out using their retention times and both UV-vis (280 and 320 nm), MS, and MS/MS spectra.

### 2.5. Determination of Antioxidant Activity

The oxygen radical absorbance capacity (ORAC) assay was carried out as proposed by Amorim, et al. [18] with modifications. Briefly, the reaction was carried out at 40 °C in black polystyrene 96-well microplates (Nunc, Denmark) using 75 mmol/L phosphate (PBS) buffer (pH 7.4) and the final assay mixture contained 120 µL fluorescein (116.7 nmol/L), 60 µL AAPH (2,2′-azobis(2-methylpropionamidine) (48 nmol/LM), and 20 µL of antioxidant (trolox standard or sample). The fluorescence was recorded for 97 min in a FluoSTAR OPTIMA microplate reader (BMG Labtech, Offenburg, Germany), with 485 nm excitation and 520 nm emission filters. The equipment was controlled by the FluoSTAR Control software version 1.32 R2 for fluorescence measurement. All reaction mixtures were prepared in duplicate and at least three independent runs were performed for each sample. The extract antioxidant activity was expressed as milligrams of Trolox equivalents per gram of leaves dry weight (mg/g DW).

### 2.6. Determination of Antimicrobial and Antibiofilm Activities

In this work *Escherichia coli* (ATCC 25922), methicillin-sensitive (MSSA) *Staphylococcus aureus* (MSSA ATCC 25923), *Salmonella enteritidis* (ATCC 13076), *Bacillus cereus* (NCTC2599), and *Listeria monocytogenes* were used as target pathogens. An inoculum of each bacteria was prepared from overnight cultures and inoculated in TSB (trypto-casein soy broth). Each lyophilized extract was re-suspended (using a vortex for vigorous agitating for ca. 5 min. If the medium still presented particles of extracts, it would be placed in a roller mixer for ca. an hour till completely dissolved) and mixed with TSB and filtered through one 0.22 µm filter to ensure that no contamination occurred.

Minimum inhibitory and bactericidal concentrations (MIC and MBC) were determined following the Clinical and Laboratory Standards Institute guidelines [19,20]. Two test solutions for each extract, at 10 and 20 mg DW /mL, were prepared and inoculated at 1% (*v*/*v*) with an inoculum of 10^8^ CFU/mL and incubated for 24 h at 37 °C. The MIC was determined by observing the lowest concentration of extract that visually inhibited bacterial growth. The MBC was determined as the lowest concentration of each extract at which bacterial growth was prevented, and the initial viability was reduced by at least 99.9% within 24 h [21]. For so, MBC was determined by inoculation on plate count agar of 20 µL aliquots of the mixtures that presented no turbidity in previous MIC determination, using the drop plate technique. All assays were performed in duplicate.

The antibiofilm activity was studied according to Silva, et al. [22] and briefly consisted on a mixture, on a 96 well microplate (Nunc, Darmstadt, Germany), of each extract with TSB at 10 and 20 mg/mL (the extracts were filtered through a 0.22 µm filter to ensure that were not contaminated) and each microorganism (ca. 1 × 10^5^ CFU/mL). The incubation was left to occur for 24 h, at 37 °C, and then the contents of the plate were discarded, each being carefully washed to remove the non-adhered cells, and the biofilms were stained using crystal violet. After shaking the microplate for 15 min, at 320 rpm, the absorbance was read at 660 nm. All assays were done in triplicate, positive control was drawn using inoculated culture media and negative control was prepared using only sterile media. The results were given as the percentage of inhibition of biofilm formation (see Equation (1))

(1) $$Antibiofilm\;activity\left(\%\right)\;=\frac{Abs\;\left(positive\;control\right)-Abs\;\left(Sample\right)}{Abs\;\left(Positive\;control\right)}\;\times \;100$$

where *Abs* (*positive control*) is the absorbance of the *positive control*, and *Abs* (*Sample*) refers to the absorbance of each sample band.

### 2.7. Determination of Extracts Capacity to Prevent/Cause DNA Oxidative Damage

The anti- and pro-oxidant capacity of the extracts was determined by the DNA assay [23], using electrophoresis to access the level of denaturation of DNA when in the presence of the extract. 

#### 2.7.1. Prevention of DNA Oxidation

The DNA solution (0.25 mg/mL, Type I (fibers) from calf thymus) was incubated in the presence of the two degradation systems selected: (i) hydrogen peroxide (H_2_O_2_) 50%, *v*/*v* and (ii) H_2_O_2_ 50%, *v*/*v* with FeCl_3_ 10 mmol/L and varying concentrations of extracts, with a range of volumes (400, 300, 200 and 100 μL), in PBS buffer. For so, the mixture of 400 μL H_2_O_2_ with y μL of sample, 400-y μL PBS and 200 μL DNA, to a final volume of 1000 μL was done (when studying the FeCl_3_ system, it were added 10 μL of FeCl_3_ 10 mmol/L, and this volume was discounted on PBS volume). The DNA solution without H_2_O_2_ was used as a positive control (no degradation) for the assays using the H_2_O_2_ system, and a DNA solution with just PBS was used as a positive control (no degradation) for the assays using the H_2_O_2_/ FeCl_3_ system. The mixture was made in duplicate for each extract. After 1 h incubation at 37 °C, in the dark, an agarose gel electrophoresis was run.

#### 2.7.2. DNA Degradation Assessment (Pro-Oxidant Assays)

The DNA solution (0.25 mg/mL) was incubated in the presence/absence of FeCl_3_ 10 mmol/L and varying concentrations of extracts, with a range of volumes (400, 300, 200, and 100 μL), in PBS buffer. For so, the mixture of y uL of the sample, 800-y uL PBS, and 200 uL DNA, to a final volume of 1000 uL was done (when studying the FeCl_3_ system, it were added 10 μL of FeCl_3_ 10 mmol/L, and this volume was discounted on PBS volume). The DNA solution without 10 mmol/L FeCl_3_ was used as a positive control (no degradation) for the assays using the FeCl_3_ system. The mixture was made in duplicate for each extract. After 1 h incubation at 37 °C, in the dark, an agarose gel electrophoresis was run. 

#### 2.7.3. Electrophoresis

Each sample was mixed 1:4 with loading buffer (25 mg bromophenol blue, 10 mL Tris EDTA (TE) buffer 1× pH 8.0, and 20 mL glycerol with pH value adjusted to 8.0) and 10 mL aliquots were transferred into a 0.75% (*w*/*v*) agarose gel prepared using TAE buffer supplemented with 0.03 mL/mL GreenSafe Premium. Electrophoresis was run for 1.25 h at 150 mV. Gels were analyzed using a molecular imager GelDOC XR+ (BioRad, Hercules, CA, USA) and the resulting image was processed using Image Lab Software v5.1 (BioRad, Hercules, CA, USA). The band area for each positive control was manually defined (band intensity) and then copied into each sample lane (maintaining the distance to the wells; with the decrease in band intensity considered to be a result of a reduction of the amount of DNA present. The results were given as the percentage of inhibition of the DNA band degradation (for the antioxidant assay) (see Equation (2)) or as a percentage of DNA band degradation (for the pro-oxidant assay) (see Equation (3))

(2) $$Inhibition\;of\;DNA\;degradation\left(\%\right)\;=\frac{Intensity\;\left(Sample\right)}{Intensity\;\left(DNA\;solution\right)}\;\times \;100$$

(3) $$DNA\;degradation\;=\;100-\;\frac{Intensity\;\left(Sample\right)}{Intensity\;\left(DNA\;solution\right)}\times \;100$$

where *Intensity* (*Sample*) is the intensity of each sample band, and Intensity (*DNA solution*) refers to the intensity of the intact *DNA solution* (positive control). 

### 2.8. Determination of Extracts Cytotoxicity and Anticancer Activity

Mucous producing human colon (HT29-MTX-E12) cells were obtained from the European Collection of Authenticated Cells Cultures (ECACC 12040401). Except when stated otherwise, the cells were grown using high glucose DMEM supplemented with 10% (*v*/*v*) heat-inactivated FBS, 1% (*v*/*v*) Pen-Strep, and 1% (*v*/*v*) of non-essential amino acids 100x. All cells were incubated at 37 °C in a humidified atmosphere with 5% CO_2_. The impact of the extract upon HT29 cell viability was determined using the XTT (2,3-bis-(2-Methoxy-4-Nitro-5-Sulfophenyl)-2H-Tetrazolium-5-Carboxanilide) colorimetric method. Briefly, aliquots of 100 µL of a cell suspension (1 × 10^5^ cell/mL) were seeded in a 96 well microplate (Nucleon Delta Surface, Thermo Scientific, Roskilde, Denmark). After seeding, the culture medium was then carefully replaced with the different test solutions and incubated in the dark. After 24 h, cell viability was assessed as follows: a 10 mmol/L of PMS solution was prepared in phosphate buffered saline (PBS, 0.01 M; pH 7.4) and a 1 mg/mL XTT solution was prepared using the appropriate culture media, previously warmed to 37 °C. Both solutions were sterilized using a 0.22 µm sterile membrane filter (Millipore, Billerica, MA, USA) and, immediately before being used, were mixed (2.5 µL of PMS per mL of XTT solution). Aliquots (25 µL) of the mixture were added to each well and the cells incubated, in the dark, for 2 h. The optical density at 485 nm was then measured using a microplate reader (FluoSTAR, OPTIMA, BMG Labtech, Ortenberg, Germany). The results were expressed as the percentage of cell metabolism inhibition.

Caco-2 (human colon carcinoma), TR146 (human squamous carcinoma), and HeLa (human cervical carcinoma) cell line cultures were kept in 75 cm^2^ T-flasks (T-75) with DMEM and incubated in a 5% CO_2_/95% air and 98% relative humidity atmosphere. When 70–80% of cells were confluent, the culture medium was removed, and the cells were rinsed with pre-warmed PBS. Cells were then detached using trypsin-EDTA, at 5% CO_2_ air atmosphere, diluted to the desired cell density (1 × 10^4^ cells per well), and left to incubate for 24 h (for HeLa and TR146 cell lines) and 48 h (for Caco-2 cell line) in a cell incubator at 37 °C in a 5% CO_2_ air atmosphere. The IC_50_ values (concentration of each extract needed to inhibit the cell metabolism in at least 50%) were calculated for TR146 cell line at passage 20–23, for HeLa cell line at passage 9–14, and Caco-2 cell line at passage 28–33. The extracts were prepared in a working solution of 200 mg/mL in dimethyl sulfoxide (DMSO) and diluted for 0.0002–2.0 mg/mL in DMEM. The negative control was prepared with 1% (*v*/*v*) Triton X-100 in DMEM; the positive control was prepared with DMEM. 200 μL of each extract was added to the wells and incubation occurred for 24 h at 37 °C in a 5% CO_2_ air atmosphere. Then the supernatant was removed, and 200 μL of MTT (3-(4,5-dimethylthiazol-2-yl)-2,5-diphenyltetrazolium bromide) solution (0.5 mg/mL) was added to each well and incubated for 4 h at 37 °C. After that, the MTT solution was discarded and replaced by 200 μL of DMSO. The mixture was shaken for 20 min at room temperature, 100–150 rpm, and the absorbance was read at 570 nm and 630 nm using a microplate spectrophotometer. After subtracting the optical density measured at 570 nm to the optical density at 630 nm (background), the impact of extracts upon the cellular metabolism was quantified accordingly to the following formula (Equation (4))

(4) $$Cell\;viability\;\left(\%\right)=\;\frac{Optical\;density\;\left(Sample\right)-Optical\;density\;\left(Negative\;control\right)}{Optical\;density\;\left(Positive\;control\right)-Optical\;density\;\left(Negative\;control\right)}\times 100$$

### 2.9. Statistical Analysis

Each parameter was studied in triplicate, being analyzed using three independent samples each time, except for cytotoxicity, for which five measurements were done for each sample (i.e., they have performed three independent replicates for each extract, each being analyzed three times, except for cytotoxicity, for which five measurements were done). Statistical analysis of the results was performed using one-way Analysis of Variance (ANOVA) followed by Tukey HSD (honestly significant difference) test, at a 5% level of significance using the Minitab Statistical Software v.17.0. The Pearson correlations were evaluated by Pearson correlation coefficient (R) and Spearman correlations were evaluated by Spearman’s correlation coefficient (rho) and the statistical significance of the coefficient (5% level of significance) using Minitab Statistical Software v.17.0. The results were expressed as mean ± standard deviation.

## 3. Results and Discussion

### 3.1. Individual Compounds

The qualitative compositional analysis of the extracts concerning phenolic compounds content was performed by LC-MS/MS and is presented in Table 2. The extracts with higher total phenolic content (determined by Folin–Ciocalteau method) were S500/20/0 and S348/20/35 (obtained by HPE, with 100% (*v*/*v*) water and 35% (*v*/*v*) ethanol, respectively), with values of 2428 ± 224 and 2343 ± 433 mg/100 g DW, respectively. All extracts obtained by HPE are composed by the same type of polyphenols; nevertheless, the control extracts lack some of the compounds, such as tuberonic acid glucoside in control aqueous and ethanolic (70% (*v*/*v*) ethanol) extracts, and sagerinic acid in all control extracts (0, 35, and 70% (*v*/*v*) ethanol). This indicates that the sagerinic acid, a common phenolic acid present in the Lamiaceae family, can be easily extracted by HPE since it is biologically present inside the cell membrane, which is damaged and destroyed by the HPE process allowing its release [24,25].

**Figure 1:**
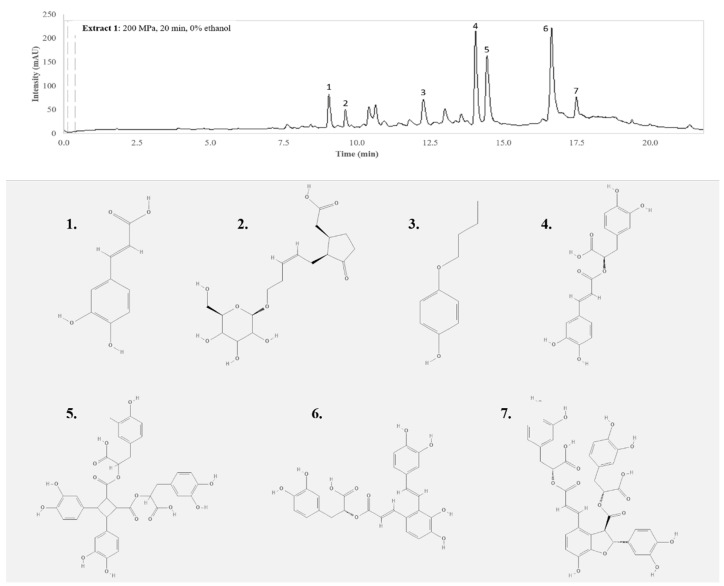
LC-MS/MS chromatogram was acquired at 320 nm of the analyses of extract 1 (S500/20/0) from winter savory leaves. The numbers 1 to 7 indicate the seven phenolic compounds identified in this extract: 1, caffeic acid; 2, tuberonic acid glucoside; 3, 4-butoxyphenol; 4, rosmarinic acid; 5, sagerinic acid; 6, salvianolic acid A; and 7, salvianolic acid B isomer.

Phenolic compounds are usually distributed in herbal extracts as a function of their polarity, so phenolic compounds are not generally found in fractions with a high amount of ethanol (non-polar fractions), while the most polar compounds remain in the water fraction [26]. Caffeic acid, rosmarinic acid, salvianolic acid A, and salvianolic acid B isomer were identified in extract 1 (S500/20/0), as also tuberonic acid glycoside, butoxyphenol, and sagerinic acid (Figure 1), which were not identified in extracts S0.1/20/0 and S0.1/1/70 (control extracts).

It is noteworthy that several peaks shown in the chromatogram could not be identified (two peaks between 10 and 11 min and another between 12.5 and 14 min). Those peaks were analyzed, but they did not match to any compound present in the available libraries or with any of the standard compounds used to create the internal library. The methods followed to try to identify those peaks were the same used for the identification of the other peaks.

To the best of the author’s knowledge, this is the first study where sagerinic acid and salvianolic acids A and B, common phenolic acids from Lamiaceae family, are reported in winter savory dried leaves extracts.

### 3.2. Antioxidant Activity

The ORAC assay is a known method that allows the measurement of the antioxidant capacity of an extract through the measurement of a fluorescent signal. Table 2 shows the antioxidant activity of the various winter savory extracts. Although for the aqueous extracts it was observed no significant differences between the HPE extract (S500/20/0) and the control at atmospheric pressure (S0.1/20/0), it is noteworthy that for the extracts S348/20/35 and S500/1/70, it was observed a clear increase of the antioxidant activity; it was obtained an increase of 37 and 73% compared to the control extracts produced with 35% (*v*/*v*) and 70% (*v*/*v*) ethanol compared with the respective extracts obtained after HPE. Furthermore, the extract S500/1/70 was the one with higher antioxidant capability using ORAC assay (679 ± 68 mg/g DW), even though it was one of the extracts presenting a relatively low concentration of total phenolic compounds (1757 ± 192 mg/g DW) when compared, for example, with the extracts S500/20/0 and S348/20/35. These results may be related to the fact that the Folin–Ciocalteau method, despite being the most used method for total phenolics determination, is susceptible to some interfering substances present in the sample, such as proteins and reducing sugars [27]. Therefore, although HPE helps to increase the extraction of components responsible for antioxidant activity, those composites are not only phenolic compounds. For example, essential oils compounds seem to be extracted using methods such as supercritical fluids, not excluding the hypothesis that HPE may help to extract some of those compounds, which could have a direct effect on the biological activities studied. According to such studies, terpenoids seems to be the most dominant components present in supercritical extracts in the range of about 50% of extraction yield [8,11,28,29]. These results are important to take into account, although essential oils were not studied in the present study, since compounds such as carvacrol, an oxygenated monoterpene, have several attributed biological activities such as antioxidant, acetylcholinesterase, and butyrylcholinesterase inhibitory, antimicrobial among others [30]. To the best of the author’s knowledge, this is the first study where antioxidant activity by ORAC assay was reported in winter savory leaves extracts.

### 3.3. Antimicrobial and Antibiofilm Activity

All extracts were subjected to antimicrobial screening by initial disk diffusion assays with 10 and 20 mg/mL, which indicated that all the extracts proved to be ineffective against the five bacterial strains tested (Gram-negative: *E. coli* and *S. enteritidis*; Gram-positive: *S. aureus*, *B. cereus*, and *L. monocytogenes*), with no zones of inhibition being observed (data not shown). MIC was initially analyzed with increasing concentrations (up to 5 mg DW/mL) and no antimicrobial activity was observed (data not shown). For so, it was performed a set of analysis where it was used only two concentrations, 10 and 20 mg of dry weight of each extract dissolved in 1 mL of TSB; 20 mg DW/mL was the highest concentration that allowed to resuspend the extracts in TSB. The MIC-values were determined by seeing the lowest concentration of extract that visually inhibited bacterial growth after a mixture of the extract with each one of the microorganisms. It was observed a MIC of 20 mg/mL for extract S348/20/35 against *L. monocytogenes* and *S. aureus*, of 20 mg/mL for extract S0.1/20/35 against *S. aureus*, and 10 mg/mL for extracts S348/20/35, S0.1/20/35, and S500/1/70 against *B. cereus* (Table 3). For both *E. coli* and *S. enteritidis,* there was observed no bacterial growth inhibition at the tested concentrations for any of the extracts studied. These results indicate that the extracts obtained after extraction using 35 or 70% (*v*/*v*) ethanol (controls or HPE ones) are richer in antimicrobial compounds (especially against Gram-positive bacteria) since the aqueous extracts presented no bacterial growth inhibition against any of the five bacterial strains, at the tested concentrations. Contrasting results were reported by Serrano, et al. [31] who obtained a MIC-value of 15.10 mg/mL against *E. coli* for ethanol extract from winter savory (obtained by maceration and stirring for 72 h, at room temperature). Additionally, the same authors reported a MIC-value of 3.00 mg/mL for *L. monocytogenes*, indicating that it is needed a much higher concentration of savory extracts to obtain bacterial growth inhibition for Gram-negative than for Gram-positive bacteria [31].

After MIC-values determination, the MBC-values were determined as the lowest concentration of each extract for which bacterial growth was prevented, by drop plate technique (Table 3). It was only observed an MBC-value of 20 mg/mL for extract S348/20/35 against *L. monocytogenes* since its initial viability was reduced by at least 99.9% within 24 h. Although other tested strains than *L. monocytogenes* are also Gram-positive, seems that the damage induced by phenolic compounds in the cell wall of *L. monocytogenes* (capable of interfering with the cell wall fluidity, possibly causing its disruption) can lead to more easy microorganism lysis [19].

Concerning the biofilm formation inhibition, it can be seen in Table 3 that generally, all extracts showed a high performance in the inhibition of biofilm formation of all the bacterial strains. Nevertheless, the extracts obtained after HPE presented better results (*p* < 0.05) than the control extracts. For both *E. coli* and *B. cereus* strains, all the extracts were able to inhibit biofilm formation, obtaining better results against *B. cereus*, since all extracts were able of >90% biofilm formation inhibition, except for S0.1/1/70 (58.97 ± 8.26%). Furthermore, for *S. aureus* and *L. monocytogenes,* the extract S0.1/20/0 (control aqueous extract) presented no activity with the lowest inhibitory effect with values of −3.82 ± 1.66 and 3.76 ± 1.04%, respectively. It was the extract S348/20/35 the one which presented better results, with values of 91.51 ± 0.61, 98.04 ± 1.41, 90.47 ± 1.06, and 96.18 ± 0.67% of biofilm formation inhibition against *E. coli*, *S. aureus*, *B. cereus*, and *L. monocytogenes*, respectively. It is interesting to note that these inhibitory effects were registered for a concentration (20 mg/mL) that was insufficient to completely inhibit microbial growth, with only a MIC at this concentration for *L. monocytogenes* and *S. aureus* being registered. These results are in accordance with the reports of several authors that demonstrated that herbal extracts (mostly the ones from Lamiaceae family) rich in phenolic compounds, such as rosmarinic acid, salvaniolic acid A and B, are able of inhibiting both Gram-negative and Gram-positive microorganisms [22,31,32,33,34]. Overall, it is interesting to note that in most cases, while exhibiting no MIC-value, the extracts were capable of inhibiting biofilm formation. This indicates that although the extracts are unable to inhibit bacterial proliferation, they still exert some impact upon the bacteria themselves. Although the reported data does not allow for a concrete hypothesis to establish as to why this occurs, some hypotheses may still be posed. An example stems from one intrinsic characteristic of phenolic compounds, namely their capacity to interact with proteins. Bacterial adhesion is frequently mediated by adhesins, membrane proteins therefore it stands to reason that should the compounds interact with these structures, it should result in an inhibition of the initial biofouling [35,36,37,38]. From a different perspective, it has become widely accepted that the establishment of a biofilm and the coordination of its lifecycle is a process that is mediated by *Quorum sensing* (QS). Therefore, another possibility for the effects of the extracts may stem from their capacity to modulate QS or other relevant metabolic pathways [39,40].

### 3.4. DNA Antioxidant Protection and Pro-Oxidant Activity

As discussed in Section 3.2 “Antioxidant activity”, there are different ROS generating systems, which can significantly affect biologically relevant molecules, such as DNA, that could result in a loss of function. In vitro, one of the means to mimic these systems uses a DNA solution (as an example of a biologically relevant molecule) in combination with (1) H_2_O_2_ and (2) H_2_O_2_ and iron cations (addition of FeCl_3_), measuring the compound/extract capacity to prevent the oxidative hydrolysis of the DNA. The combination of H_2_O_2_ and FeCl_3_ leads to an induction of more extensive damage to DNA than H_2_O_2_ alone because the presence of cationic iron will stimulate the decomposition of peroxide into hydroxyl and peroxyl radicals will have higher reactivity than peroxide alone. The six different extracts were tested at a maximum concentration of 5.0 mg DW/mL applied to a mixture combining DNA solution and a ROS generating system. From this assay, two biological properties can be obtained, the antioxidant activity (the measurement of the level of DNA degradation inhibition), and the pro-oxidant activity (the measurement of the level of DNA degradation caused by the extract itself) [23].

**Figure 2:**
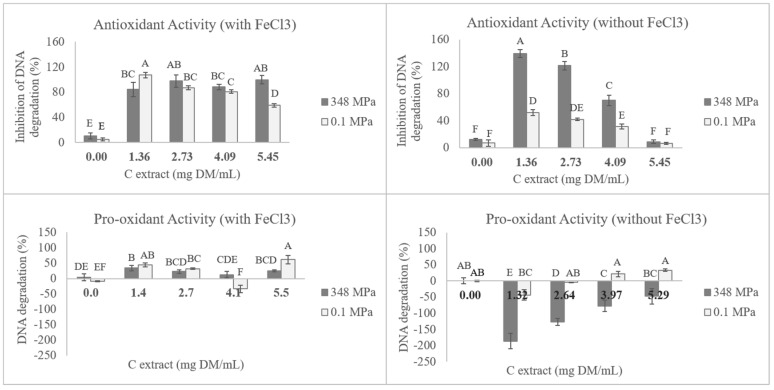
Antioxidant activity, i.e., prevention of DNA degradation (in an H_2_O_2_ system or and H_2_O_2_/FeCl_3_ system). Pro-oxidant activity (in the presence and absence of iron cations) for 35% (*v*/*v*) ethanol extracts (extracts S348/20/35 and S0.1/20/35). Different letters indicate significant differences (*p* < 0.05) between extracts for each condition set.

The efficiency of the different extracts in preventing oxidative damage of DNA induced by H_2_O_2_ (antioxidant activity) was evaluated. In Table 4 it is possible to see that the ability of the several extracts to protect the DNA molecule is dependent on the presence/absence of iron cations in the reaction since for the higher concentration studied, all extracts demonstrated higher DNA oxidation inhibition in the presence of iron than in its absence. Although it was the extract S500/1/70 the one that presented higher (*p* < 0.05) antioxidant activity (124.4 ± 13.31% inhibition of DNA degradation) at the higher concentration (5.0 mg DW/mL), it was the extract S348/20/35 the one with better results (*p* < 0.05) at lower concentrations (Table 4, Figure 2), indicating that it is possible to obtain extracts by HPE with high DNA protective effect, even at low concentrations (~1.0 mg DW/mL), assuring to obtain a value of 138.9 ± 5.8 and 84.5 ± 11.4% inhibition of DNA degradation in the absence and presence of FeCl_3_, respectively. This hints that this extract presents a better balance in quenching both highly reactive radicals (hydroxyl and peroxyl) as well as less reactive ones (peroxide).

Concerning the pro-oxidant assay (direct DNA damage effect), it is noteworthy that all the control extracts (S0.1/20/0, S0.1/20/35, and S0.1/1/70) presented higher pro-oxidant activity (from 31.2 ± 6.4 to 76.2 ± 10.6% of DNA degradation) than the extracts obtained after HPE (S500/20/0, S348/20/35, and S500/1/70), which presented negative values from −16.4 ± 13.7 to 28.0 ± 4.1% of DNA denaturation. These results indicate that in the case of the HPE extracts, there was no relevant interaction between the compounds inducing DNA oxidation, meaning that HPE extracts can protect the DNA molecule, with no damaging capacity even at high concentrations. To the best of the author’s knowledge, this is the first study where antioxidant activity by DNA damage protective effect was reported in winter savory extracts.

It is interesting to note that in the assay in the absence of iron cations, the degradation inhibition percentages in the antioxidant assay were above 100%. Mathematically speaking this occurred because the intrinsic fluorescence of bands in the presence of the extract was higher than the bands by themselves. Silva, Costa, Vicente, Veiga, Calhau, Morais and Pintado [23] proposed that this could be a consequence of interactions between the DNA molecule and the phenolic compounds present in the extract that by altering the coiling of the DNA it could also alter the fluorescent die capacity to interact with it. The hypothesis that is also supported by the work of Kanakis, et al. [41], in which the presence of phenolic compounds was described as interacting with the major and minor grooves of the DNA structure as well as with the phosphate backbone in a manner that affected its physical- and electrochemical properties. Moreover, when looking at the pro-oxidant assay a similar effect appears to have occurred, i.e., the negative degradation percentage, which implies a stronger fluorescence than that of the original DNA control, with this effect being stronger in the 348 MPa extract. Additionally, it is interesting to note that this likely interaction between the extract and the DNA molecule is not observed in the presence of Fe^3+^. The compounds may mediate the cation reduction, resulting in a modification of its reactivity and capacity to interact with DNA.

### 3.5. Cytotoxicity

The mucus-secreting HT29-MTX intestinal cell line was used to study the possible cytotoxic effect of winter savory extracts using the XTT cell viability assay, by measurement of cell number based on metabolic activity, since, when confluent, these cells mimic human cell behavior. The results demonstrated that the extracts at 0.5 mg DW/mL did not exert any inhibition of the cellular metabolism, appearing, to stimulate it (negative values of metabolism inhibition), which could mean that the presence of the extracts could induce an increase of the metabolic rate of HT29-MTX cell line, and for that demonstrating the absence of cytotoxicity. Only the extract S0.1/1/70 presented an inhibition above 20% (22.0 ± 2.0%), indicating that this extract could cause some inhibition of the cellular development, but also with relevance since values are lower than 30% cell metabolism inhibition. When testing the concentration of 1.0 mg DW/mL, it was observed that all control extracts led to metabolism inhibition above 30% (although very close to the limit [42]), while HPE extracts did not induce significant cell metabolism inhibition of intestinal cells (up to 30%), so demonstrating no cytotoxic effect (Table 4).

**Figure 3:**
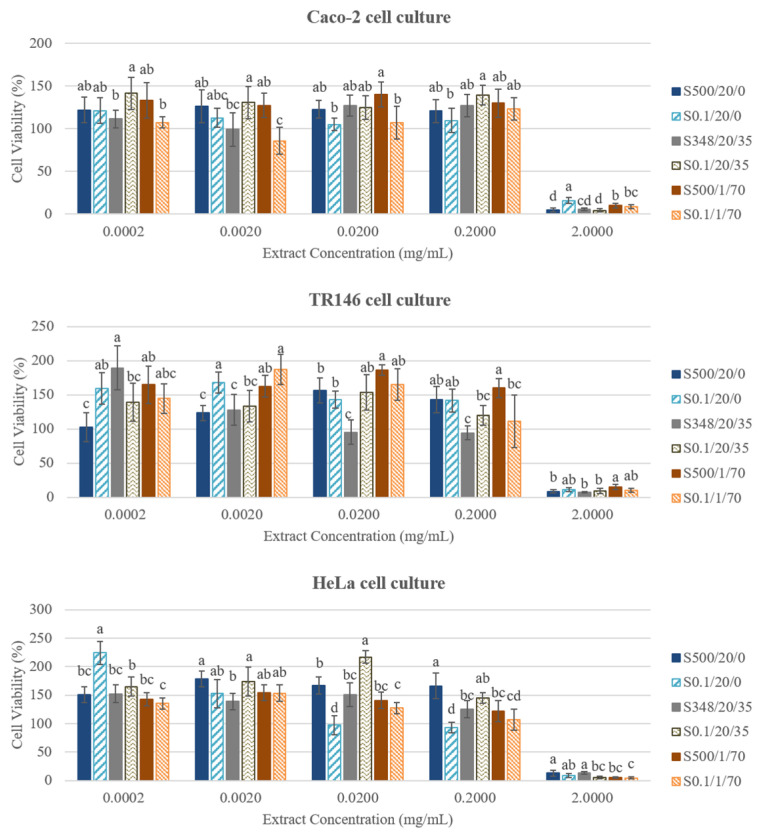
Results of cell viability in percentage, obtained for the six extracts of *S. montana* with Caco-2, TR146, and HeLa cell culture lines. Different letters indicate significant differences (*p* < 0.05) between extracts for each concentration studied (0.0002 to 2.000 mg/mL).

To evaluate the potential for antitumoral activity, some carcinogenic cell lines were exposed to the extracts. For so, cell lines such as Caco-2 cell line from heterogeneous human epithelial colorectal adenocarcinoma, TR146 cell line from human squamous carcinoma, and HeLa cell line from cervical cancer, were also studied as cellular models to evaluate the cytotoxic effect of extracts. The extracts were tested in a concentration ranging from 0.0002 mg/mL to 2.0 mg/mL, and it was possible to see that the higher concentration, 2.0 mg/mL of all extracts (HPE and controls), led to cell viability values below of 15% for the three cell lines, confirming high inhibitory effect, while lower concentrations (0.2 mg/mL and below) led to cell viability values equal to higher than 100% (Figure 3).

Moreover, extracts exhibited antiproliferative effect on Caco-2, TR146, and HeLa cell lines with IC_50_ values ranging from 0.756–1.932 mg/mL, 0.669–1.795 mg/mL, and 0.629–1.715 mg/mL, respectively. HeLa cell line proved to be the most sensitive one since lower concentrations of the extracts were needed to inhibit cell survival by 50%. Similar results were obtained by Četojević-Simin, et al. [43], who reported the effect of winter savory extracts pretreated with 70% (*v*/*v*) methanol on HeLa, HT-29, and MCF-7 cell lines. These authors also observed that HeLa cell line is the most susceptible, with IC_50_ values ranging from 0.41 mg/mL (extract using ethyl acetate) to 0.84 mg/mL (aqueous extracts). Also, Elgndi, Filip, Pavlić, Vladić, Stanojković, Žižak and Zeković [10] demonstrated that HeLa cells are the most sensitive, obtaining values of IC_50_ ranging from 0.88 to 0.91 mg/mL with extracts obtained by supercritical CO_2_ extraction, and reporting that essential oil from winter savory is about 1.5 times more cytotoxic (IC_50_ of 0.60 mg/mL) than the extracts obtained by supercritical CO_2_ extraction after 4 h. Several studies have been reported that the major components of essential oil from winter savory (carvacrol and thymol, representing about 50% of the oil) are highly cytotoxic against human metastatic cancer cells, indicating that these components may be used as potent therapeutic in the treatment of cancer [10,44,45,46]. Therefore, it could be due to these kinds of compounds (not studied in the present work) that HPE extracts present some cytotoxic activity against the carcinogenic cell lines.

Furthermore, it should be noted that extracts rich in phenolic compounds can present high pro-oxidant activity even at low concentrations since there is a possibility of polyphenol-mediated H_2_O_2_ formation when present in culture media. This can lead to the production of significant amounts of reactive oxygen species (such as H_2_O_2_), which can lead to cellular gene expression modulation, or even apoptosis [47]. Bellion, Olk, Will, Dietrich, Baum, Eisenbrand and Janzowski [47] reported a high H_2_O_2_ production by extracts rich in polyphenols under growing conditions commonly used for HT-29 cell lines development. These results indicate those extracts can effectively produce H_2_O_2_ when in cell culture medium DMEM, even without cells, increasing its levels with time and extract concentration, which is a factor to consider when studying cytotoxicity and pro-oxidant activities by herbal extracts [47].

### 3.6. Correlation between Variables

A correlation coefficient aims to evaluate the extent to which two variables tend to change together, by describing both the strength and the direction of those relationships. Table 5 shows two different correlations: the Pearson correlation (R-value) that evaluates if the two variables have a linear relationship, i.e., the variables change proportionally to one another; while the Spearman correlation coefficient (rho-value) measures the potential non-linear relationship between two variables, i.e., the variables tend to change together, but not necessarily proportionally.

In Table 5, it is possible to see that antioxidant activity analyzed by ORAC assay has no significant correlation (linear nor non-linear) to any of the other variables under study, which corroborates the hypothesis that although HPE helps to increase the extraction of components responsible for antioxidant activity, the compounds responsible for the antioxidant activity are probably not only derived from phenolic compounds. As stated before, the compounds of the essential oils (ex. tripernoids) seem to be extracted using pressurized methods such as supercritical fluids, with it being possible that HPE may help to extract some of those compounds, which would have a direct impact on the bioactivities studied here. Nonetheless, the antioxidant activity measured by DNA assay, presented a high rho-value for total phenolic compounds, probably due to the presence of tuberonic acid glucoside, the isomer of salvianolic acid B, 4-butoxyphenol, sagerinic acid, and salvianolic acid A, since those phenolic acids were already reported to act as cell protectors against oxidative stress by several authors [48,49,50].

## 4. Conclusions

*Satureja montana* L. has been majorly studied concerning its essential oils and its bioactivities. The present study allowed us to study with more detail the several biological properties related to the diverse composition of secondary metabolites from its leaf extracts. In conclusion, the extracts obtained by HPE exhibit a relevant range of biological activities, particularly the extract S348/20/35, obtained at 348 MPa, 20 min, using 35% (*v*/*v*) ethanol that proved to be the extract with higher potential as an antioxidant, DNA protection, with higher ability to inhibit biofilm formation of *E.coli, S. aureus, B. cereus,* and *L. monocytogenes*, and was also the only extract that showed a value of MBC of 20 mg/mL against *L. monocytogenes*. The extracts obtained by HPE also presented a higher antioxidant activity than controls, either measured by ORAC assay or by DNA degradation assay, both biological important methods. It is noteworthy that winter savory extracts, especially the ones obtained after HPE, were able not only to not induce significant damage in the DNA molecules compared to the controls, as were also able to protect it against the damage caused by oxidative stress in the presence of ROS. Concerning the cytotoxicity of the extracts, it was observed that HPE extracts, in a concentration of 0.5 mg/mL, were not considered harmful to HT29 cell lines (a cell culture commonly used to assess the biocompatibility of extracts to human intestinal cells). Nevertheless, in a higher concentration (>1.0 mg/mL), although HPE extracts do not reveal significant inhibition, the control extracts demonstrate a slight significant reduction of the cell viability.

The results reported in the present work, bring some insight into the potential biological activities of winter savory leaves extracts. Nevertheless, it is necessary to perform advanced analysis concerning the extracts bioavailability and if the studied bioactivities remain noteworthy in in vivo studies.

## Figures and Tables

**Table 1:** Identification of extracts according to the conditions of pressure, extraction time, and ethanol concentration used as a solvent to perform HPE.

	Pressure Level	Extraction Time	Solvent	Nomenclature
Extract 1	500 MPa	20 min	0% (*v*/*v*) Ethanol	S500/20/0
Extract 2	0.1 MPa	20 min	0% (*v*/*v*) Ethanol	S0.1/20/0
Extract 3	348 MPa	20 min	35% (*v*/*v*) Ethanol	S348/20/35
Extract 4	0.1 MPa	20 min	35% (*v*/*v*) Ethanol	S0.1/20/35
Extract 5	500 MPa	1 min	70% (*v*/*v*) Ethanol	S500/1/70
Extract 6	0.1 MPa	1 min	70% (*v*/*v*) Ethanol	S0.1/1/70

**Table 2:** Phenolic compounds identified in winter savory leaves extracts by LC-MS/MS, and antioxidant activity obtained by ORAC assay. Different letters indicate significant differences (*p* < 0.05) between extracts for each condition set (differences analyzed by column).

Identification of Extracts	Total Phenolic Compounds (g/100 g DW)	Caffeic Acid	Tuberonic Acid Glucoside	4-Butoxyphenol	Rosmarinic Acid	Sagerinic Acid	Salvianolic Acid A	Salvianolic Acid B Isomer	Antioxidant Activity (mg_Trolox_/g DW)
S500/20/0	2.4 ± 0.2 a	✓	✓	✓	✓	✓	✓	✓	(4.1 ± 0.5) × 10^2^ c
S0.1/20/0	1.2 ± 0.9 c	✓	n.d.	✓	✓	n.d.	✓	✓	(4.1 ± 0.8) × 10^2^ c
S348/20/35	2.3 ± 0.4 ab	✓	✓	✓	✓	✓	✓	✓	(5.4 ± 0.6) × 10^2^ b
S0.1/20/35	2.0 ± 0.4 ab	✓	✓	✓	✓	n.d.	✓	✓	(3.9 ± 0.2) × 10^2^ c
S500/1/70	1.8 ± 0.2 bc	✓	✓	✓	✓	✓	✓	✓	(6.8 ± 0.7) × 10^2^ a
S0.1/1/70	1.2 ± 0.3 c	✓	n.d.	n.d.	✓	n.d.	✓	✓	(3.9 ± 0.4) × 10^2^ c

Note: n.d. means that the compound was not detected; ✓ means that the phenolic compound was identified in the extract.

**Table 3:** Minimum inhibitory concentration (MIC), minimum bactericidal concentration (MBC), and biofilm formation inhibition capacity of winter savory leaves extract against pathogenic bacteria commonly found in food products. Different letters indicate significant differences (*p* < 0.05) between extracts for each assay (analysis by column).

	Identification of Extracts	*E. coli*	*S. enteritidis*	*S. aureus*	*B. cereus*	*L. monocytogenes*
MIC (mg/mL)	S500/20/0	n.d.	n.d.	n.d.	n.d.	n.d.
	S0.1/20/0	n.d.	n.d.	n.d.	n.d.	n.d.
	S348/20/35	n.d.	n.d.	20	10	20
	S0.1/20/35	n.d.	n.d.	20	10	n.d.
	S500/1/70	n.d.	n.d.	n.d.	10	n.d.
	S0.1/1/70	n.d.	n.d.	n.d.	n.d.	n.d.
MBC (mg/mL)	S500/20/0	>20	>20	>20	>20	>20
	S0.1/20/0	>20	>20	>20	>20	>20
	S348/20/35	>20	>20	>20	>20	20
	S0.1/20/35	>20	>20	>20	>20	>20
	S500/1/70	>20	>20	>20	>20	>20
	S0.1/1/70	>20	>20	>20	>20	>20
Biofilm formation inhibition (%)		***E. coli***	***S. enteritidis***	***S. aureus***	***B. cereus***	***L. monocytogenes***
	S500/20/0	89.05 ± 0.11 b	81.37 ± 5.05 a	11.75 ± 2.39 d	98.42 ± 1.43 a	89.60 ± 2.68 a
	S0.1/20/0	81.75 ± 1.20 c	56.98 ± 10.26 b	−3.82 ± 1.66 e	96.64 ± 2.35 a	3.76 ± 1.04 c
	S348/20/35	91.51 ± 0.61 b	42.02 ± 9.80 bc	98.04 ± 1.41 a	90.47 ± 1.06 a	96.18 ± 0.67 a
	S0.1/20/35	69.02 ± 3.23 d	12.44 ± 3.22 de	93.51 ± 1.55 ab	89.92 ± 6.73 a	93.05 ± 6.02 a
	S500/1/70	97.62 ± 0.60 a	35.19 ± 6.85 cd	91.03 ± 1.63 b	90.04 ± 1.46 a	93.01 ± 7.05 a
	S0.1/1/70	81.68 ± 1.42 c	5.00 ± 0.51 e	41.16 ± 3.49 c	58.97 ± 8.26 b	74.20 ± 8.31 b

Note: Results presented only for the higher concentrations (in mg DW/mL) tested for each extract: S500/20/0: 20.10; S0.1/20/0: 20.48; S348/20/35: 19.94; S0.1/20/35: 20.25; S500/1/70: 20.47; S0.1/1/70: 20.08. n.d. means that the activity was not detected.

**Table 4:** Results for DNA extraction, degradation (expressed in percentage of DNA degradation inhibition for antioxidant activity and percentage of DNA degradation for pro-oxidant activity) and cytotoxicity (expressed in percentage of cell metabolism inhibition for HT29-MTX; and in IC_50_ (mg/mL) for Caco-2, TR146, and HeLa cell lines). Different letters indicate significant differences (*p* < 0.05) between extracts for each assay (analysis by column).

	Antioxidant Activity (%) *		Pro-Oxidant Activity (%) *		Cytotoxicity				
	With FeCl_3_	Without FeCl_3_	With FeCl_3_	Without FeCl_3_	HT29-MTX		Caco-2	TR146	HeLa
					(%cell Metabolism Inhibition)		(IC_50_ (mg/mL))		
					0.5 mg/mL	1.0 mg/mL			
S500/20/0	73 ± 17 bc	65 ± 11 a	−16 ± 14 c	−14 ± 7 d	−25 ± 2 c	24 ± 3 d	1.47	1.50	0.63
S0.1/20/0	68 ± 5 c	36 ± 7 b	31 ± 6 b	14 ± 4 c	14 ± 7 a	38 ± 0 a	1.59	1.61	0.67
S348/20/35	100 ± 7 ab	9 ± 3 cd	25 ± 6 b	−46 ± 23 e	−22 ± 5 c	30 ± 2 bc	1.64	0.67	0.85
S0.1/20/35	59 ± 3 c	6 ± 2 d	62 ± 1 a	34 ± 4 bc	−8 ± 2 b	38 ± 2 a	0.76	0.71	1.51
S500/1/70	124 ± 13 a	22 ± 3 c	28 ± 4 b	46 ± 6 ab	13 ± 3 a	25 ± 1 cd	1.93	1.80	1.72
S0.1/1/70	73 ± 17 c	20 ± 5 cd	76 ± 11 a	63 ± 12 a	22 ± 2 a	32 ± 1 b	1.49	1.57	1.53

* Results presented only for the higher concentrations (in mg DW/mL) tested for each extract: S500/20/0: 5.42; S0.1/20/0: 5.39; S348/20/35: 5.45; S0.1/20/35: 5.28; S500/1/70: 5.20; S0.1/1/70: 5.15.

**Table 5:** Pearson (*R*-value) and Spearman (rho-value) correlations and respective *p*-value in parenthesis. It should be noted that the table is labeled with a color code (only the significant correlations (*p* < 0.05) are highlighted in grey cells, and more intense color represents the highest correlation values (three levels: light grey: *R*-value below 0.650; medium grey: *R*-value from 0.650 to 0.750; dark grey: *R*-value above 0.750).

			DNA Degradation (%)		Inhibition of DNA Degradation (%)		Biofilm Formation Inhibition (%)					Cytotoxicity (% Cell Viability)			
		ORAC (mg/g)	Without Fe^3+^	With Fe^3+^	Without Fe^3+^	With Fe^3+^	*E. coli*	*S. enteritidis*	*S. aureus*	*B. cereus*	*L. monocytogens*	HT29-MTX	Caco−2	TR146	HeLa
Pearson Correlation (R)	Phenolics	0.376 (0.206)	−0.638 (0.010)	−0.524 (0.045)	−0.431 (0.084)	0.044 (0.862)	0.273 (0.273)	0.307 (0.248)	0.331 (0.179)	0.458 (0.056)	0.656 (0.004)	−0.876 (0.000)	−0.837 (0.022)	−0.806 (0.013)	0.693 (0.016)
	ORAC	n.d.	−0.381 (0.145)	−0.064 (0.813)	−0.267 (0.285)	0.776 (0.000)	0.421 (0.225)	−0.045 (0.908)	0.538 (0.108)	−0.048 (0.896)	0.474 (0.197)	0.382 (0.198)	−0.006 (0.978)	0.117 (0.596)	0.154 (0.433)
Spearman correlation (rho)	Phenolics	0.264 (0.384)	−0.579 (0.024)	−0.568 (0.027)	−0.348 (0.171)	0.848 (0.049)	0.375 (0.125)	0.312 (0.239)	0.325 (0.188)	0.349 (0.156)	0.551 (0.022)	−0.862 (0.000)	−0.296 (0.266)	−0.529 (0.043)	0.499 (0.069)
	ORAC	n.d.	−0.412 (0.113)	−0.168 (0.535)	−0.168 (0.505)	0.717 (0.001)	0.491 (0.150)	0.000 (1.000)	0.285 (0.425)	−0.467 (0.174)	0.267 (0.488)	−0.341 (0.255)	0.019 (0.926)	−0.150 (0.494)	0.234 (0.230)

## Supplementary Materials

Supplementary File 1
