# Supplementary material for: Effect of High Hydrostatic Pressure Extraction on Biological Activities and Phenolics Composition of Winter Savory Leaf Extracts
Source: Antioxidants (Basel). 2020 Sep 8;9(9):841. doi: 10.3390/antiox9090841 (PMC7554779; doi:10.3390/antiox9090841)
Supplement: Supplementary File 1 [file antioxidants-09-00841-s001.pdf]

Table S1. Phenolic compounds identified and quantified in winter savory leaves extracts by LC-MS/MS.

	**Caffeic acid**	**Tuberonic acid glucoside**	**4-Butoxyphenol**	**Rosmarinic acid**	**Sagerinic acid**	**Salvianolic acid A**	**Salvianolic acid B isomer**
Formula	$C_9H_8O_4$	$C_{18}H_{28}O_9$	$C_{10}H_{14}O_2$	$C_{18}H_{16}O_8$	$C_{36}H_{32}O_{16}$	$C_{26}H_{22}O_{10}$	$C_{36}H_{30}O_{16}$
$[M-H]^-$ (m/z experimental)	179.0346	387.1654	165.0915	359.0765	719.1608	493.1136	717.1446
$[M-H]^-$ (m/z calculated)	179.0350	387.1661	165.0921	359.0772	719.1618	493.114	717.1461
MS/MS fragments	135.0444 (100)<br>179.0345 (22.6)	387.1656 (100)<br>207.1023 (35.90)<br>119.0344 (11.22)	165.0914 (100)<br>164.0834 (70.80)<br>149.0596 (36.85)<br>122.0362 (6.53)	161.0240 (100)<br>197.0453 (73.67)<br>179.0343 (24.86)	161.0238 (100)<br>359.0770 (84.31)<br>197.0453 (37.66)<br>179.0343 (4.32)	161.0236 (100)<br>359.0767 (42.60)<br>197.0451 (38.47)<br>135.0443 (32.73)	519.0916 (100)<br>339.0506 (91.97)<br>135.0443 (36.14)
